# The importance of tissue science and valve design in relation to durability and hemodynamics of the DurAVR aortic heart valve

**DOI:** 10.3389/fcvm.2025.1512961

**Published:** 2025-02-07

**Authors:** William M. L. Neethling, Guenther Forster, Christopher Meduri, Bart Meuris, Anita W. Asgar, Stephanie Sellers, João L. Cavalcante, Vinayak Bapat, Michael Reardon

**Affiliations:** ^1^ Cardiovascular Research, Anteris Technologies Ltd, Perth, WA, Australia; ^2^ Department of Cardiology, Karolinska University Hospital, Stockholm, Sweden; ^3^ Cardiac Surgery, University Hospitals Leuven, Leuven, Belgium; ^4^ Structural Heart Program, Institut de Cardiologie de Montreal, Montreal, QC, Canada; ^5^ Cardiovascular Translational Laboratory, Providence Research & Centre for Heart Lung Innovation, University of British Columbia, Vancouver, BC, Canada; ^6^ Section of Cardiac Imaging, Allina Health Minneapolis Heart Institute at Abbott Northwestern Hospital, Minneapolis, MN, United States; ^7^ Department of Cardiac Surgery, Allina Abbott Northwestern Hospital, Minneapolis, MN, United States; ^8^ Department of Cardiothoracic Surgery, Houston Methodist Hospital, Houston, TX, United States

**Keywords:** structural valve deterioration, calcification, ADAPT^TM^, hemodynamics, TAVR, bioprosthetic valve

## Abstract

**Introduction:**

Clinical evidence highlighting the efficacy and safety of transcatheter aortic valve replacement (TAVR) and the 2019 Food and Drug Administration (FDA) approval for TAVR in low-risk (younger) patients has created a demand for durable and long-lasting bioprosthetic heart valve (BHV) leaflet materials. Over the life of an implanted BHV mechanical stress, immunogenicity, calcification, and hemodynamic dysfunction lead to failure via structural valve deterioration (SVD). Consequently, the durability of the bioprosthetic materials selected for valve manufacture is of utmost importance.

**Technology:**

The ADAPT™ tissue engineering process, an anti-calcification preparation that transforms xenograft tissue (bovine pericardium) into a durable valve bioscaffold, shows significant clinical benefits in mitigating the interrelated mechanisms leading to SVD. The novel acellular, biostable and non-calcifying biomaterial has recently been molded into a single-piece 3D biomimetic valve (DurAVR™) with excellent early clinical results and the potential to meet the growing demand of durable BHVs for the treatment of aortic stenosis.

**Discussion:**

The unique design of the DurAVR biomimetic valve in combination with the superior biostability of ADAPT tissue could advance the BHV space by providing superior performance and durability to aortic stenosis patients in need of TAVR.

## 1 Introduction

Transcatheter aortic valve replacement (TAVR) was developed as a treatment option for patients with aortic stenosis (AS) and a high-risk for open-heart surgery. In 2019, as the annual number of TAVR cases began to exceed surgical cases (1), continued improvements in procedural clinical efficacy and safety led to the approval of TAVR for younger, lower-risk patients by the US Food and Drug Administration (FDA). The COVID-19 pandemic in 2020 caused a shift toward less invasive treatment options intended to shorten in-hospital stays without compromise of short-term outcomes (2). More recently, the addition of positive 4- and 5-year data from randomized controlled trials comparing TAVR to surgical implants in patients with a low-risk for open-heart surgery (3, 4) suggests that there will continue to be a dramatic increase in the yearly caseload of TAVR procedures in the coming years (5).

As an alternative to mechanical heart valves, the use of bioprosthetic heart valves (BHV) is gaining popularity with several key advantages. However, the biomaterials used in current BHV designs have yet to be optimized to attenuate structural valve deterioration (SVD) and remain fully functional for the patient's expected lifespan. The market for novel biologics and tissue engineering processes has expanded within the last 10 years, which in turn, has greatly enhanced valve leaflet integrity. However, the need for the development of a valve that achieves true for long term durability remains elusive and, the need for repeat procedure such as valve in valve or redo-TAVR poses a significant public health issue.

The ADAPT™ tissue engineering process is an anti-calcification preparation that transforms xenograft tissue, such as bovine serum albumin (BSA)-free bovine pericardium, into a durable bioscaffold that has the potential to mitigate SVD. The novel, acellular, biostable, and non-calcifying biomaterial has recently been molded into a single-piece 3D tissue heart valve to meet the growing demand of durable bioprosthetic for TAVR. Moreover, clinical data at 1 year shows sustained hemodynamic performance with no signs of SVD (6). This scientific review focuses on the evolution of the ADAPT tissue engineering process and explores its application to a novel 3D single-piece biomimetic tissue heart valve (DurAVR™).

## 2 Aortic valve disease, treatments, and structural valve deterioration

### 2.1 Aortic valve structure and function

**Figure 1:**
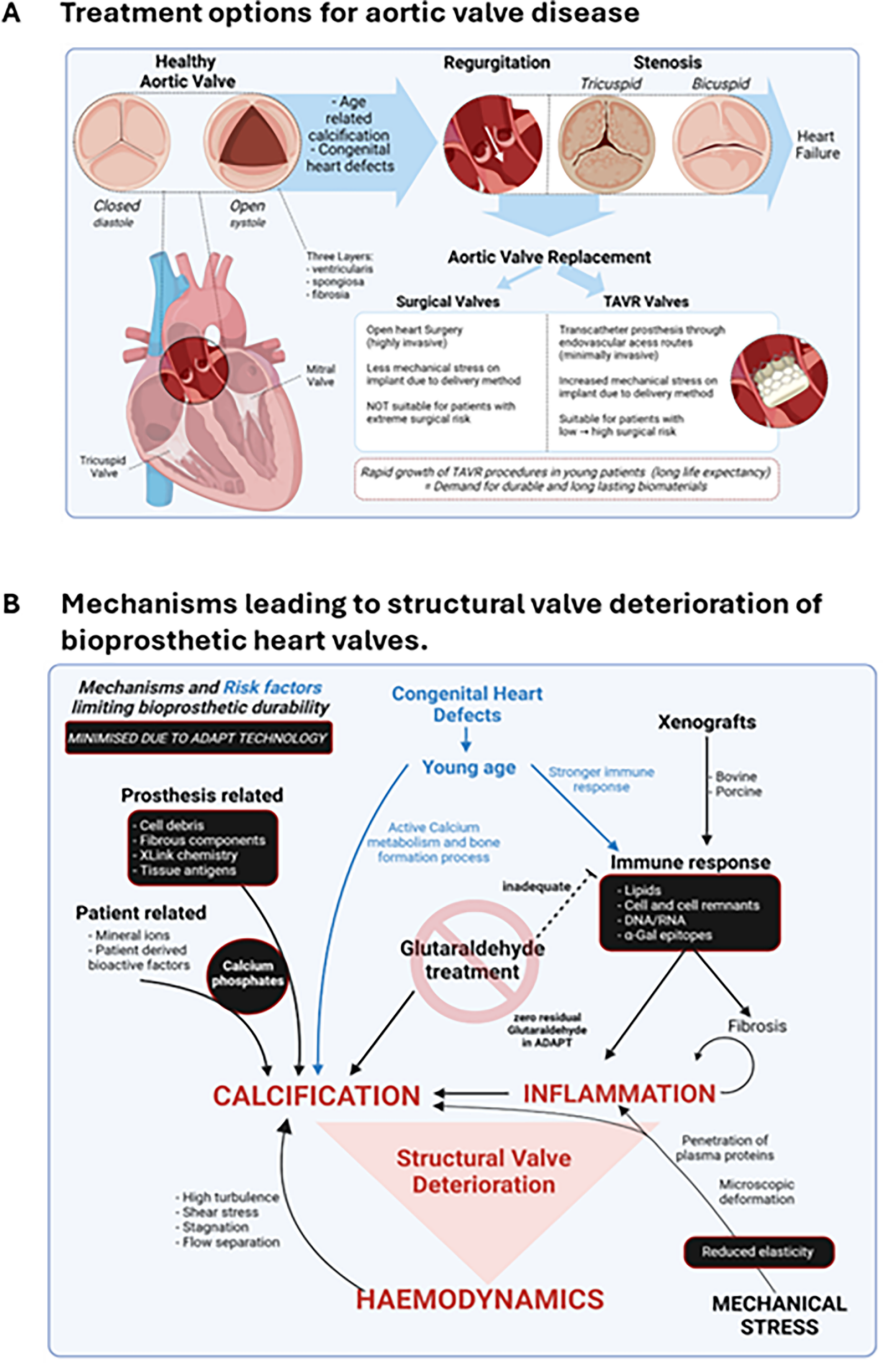
Aortic valve disease. **(A)** Age-related calcification and congenital heart defects can lead to severe aortic valve conditions including regurgitation and stenosis. If left untreated, conditions can lead to heart failure with increased mortality rates. Patients with aortic valve disease typically have two treatment options, surgical valve replacement or TAVR. **(B)** SVD occurs due to several interrelated mechanisms that increase inflammation, calcification, and impair valve hemodynamics. Contextual factors such as a young host with a strong immune response can increase the risk of developing SVD. The origin of the biomaterial can also carry added risk, with xenografts from bovine or porcine origin being recognized by the host as a foreign tissue. In addition, commonly applied chemical glutaraldehyde treatment of the implanted material is ineffective at removing cellular antigens and can exacerbate calcification. The ADAPT™ tissue engineering process aims to mitigate or abolish key risk factors leading to SVD (identified in black boxes). TAVR, transcatheter aortic valve replacement; SVD, structural valve deterioration.

The aortic valve (AV) generally presents with three cusps composed of collagen, elastin, glycosaminoglycans and proteoglycans arranged in a complex layered configuration (Figure 1A). This composite structure provides both flexibility for dynamic opening and closing and tensile strength to resist transvalvular pressure (7). Due to its role dividing a major pressure differential, the AV is subject to significant physical wear and tear. As such, when valve function is impaired via stenosis and/or regurgitation, valve repair or replacement is necessary to avoid heart failure.

### 2.2 Bioprosthetic heart valves

Cardiac surgeons and interventional cardiologists have multiple options when choosing a bioprosthetic heart valve. Reduced coagulation concerns and recent developments in tissue engineering processes have increased BHV longevity making them a favorable choice over their mechanical counterparts. The differing tissue sources, preparation procedures and valve geometries of commercially available BHV dramatically influence the durability and biostability of the implanted valve. The innate elastic properties and uniform suture retention of xenogeneic pericardium derived from both bovine and porcine sources has been incorporated in many BHV leaflet designs. However, despite effective tissue processing techniques designed to mitigate damage and host immune response to the prosthesis, commercial valves and patches derived from xenogeneic tissue are still at risk of failure due to calcification, infection, and thrombo-embolic events (8).

### 2.3 Structural valve deterioration

Mechanisms underlying structural valve deterioration (SVD) remain incompletely understood and additional research to fully elucidate the degenerative process are needed. Durability is measured as an outcome of resistance to SVD, irreversible multifaceted process resulting in impaired structure and function of the valve (Figure 1B). In addition to the alternating mechanical stresses generated by the challenging hemodynamic environment and the cyclic opening and closing demands on the BHV, there are multiple biologic pathways that can initiate SVD.

### 2.4 Biologic mechanisms of structural valve deterioration

The presence of xenogeneic antigens such as cells and cell remnants, nucleic acids, and lipids in BHV leaflets trigger an immune response resulting in excessive fibrosis and calcification mediated SVD (9, 10) (Figure 1B). This is particularly true for galactose-α-1,3-galactose (α-Gal) epitopes, which are carbohydrate antigens expressed in animal derived tissues, as well as residual DNA and RNA that trigger a cytokine storm and play a key role in immune-mediated biological heart valve failure (11, 12).

Damage to the native tissue inflicted during the implantation procedure can also exacerbate the foreign body reaction by adhesion of serum proteins to the surface of the implant triggering the contact activation system, fibrinolysis and complementary cascades, resulting in adhesion of platelets and activated leukocytes to the surface of the BHV (13). Platelet and leukocyte adhesion in the peri-implant area causes inflammation and thrombosis. This results in the emergence of immune infiltrates and leads to fibrovascular outgrowths, also known as pannus, in areas where recipient tissue and the implant make contact (14).

Driven by factors related to host metabolism, implant tissue structure, physical stress, and implant chemistry, calcification is considered to be the primary cause of SVD in BHV (15) (Figure 1B). Other known calcification agents include membrane-associated complexed acidic phospholipids and xenogeneic cell component biomaterials (i.e., extracellular matrix proteins, non-viable interstitial cells and cell remnants). The intrinsic chemical properties of the leaflet are known to either promote or inhibit calcification depending on the tissue engineering process (8).

### 2.5 Efficacy of common tissue fixation processes in mitigating structural valve disease

Glutaraldehyde treatment of bovine pericardium, proposed as an effective way to remove *α*-Gal and maintain durability through the cross-linkage of collagen molecules, has been shown to reduce the antigenic response to BHV leaflets (16, 17). However, glutaraldehyde is reported to increase the rigidity of the BHV leaflet, which results in increased destruction of collagen and elastin fibers during cyclic deformation (13). In addition, glutaraldehyde-treated BHV leaflets are susceptible to calcification, especially in younger patients (18). It is thought that glutaraldehyde fixation exposes aldehyde groups that react with circulating phospholipids and calcium ions (19). This fixation approach also removes soluble proteins (i.e., glycoproteins) from the tissue that are reported to block calcium-binding sites and thereby inhibit the start of the calcification process (20). Moreover, decellularization processes have also been proposed as an effective way to remove all cell remnants from the bioprosthetic material. However, this process leaves trace amounts of nucleic acids, an extremely potent inducer of a negative immune response to the implanted BHV (21).

### 2.6 Hemodynamic impact of structural valve deterioration

Each mechanism of SVD outlined in Figure 1B impacts the hemodynamic function of the implanted BHV. It has been well documented that the disruption of blood flow through the BHV leads to both high turbulence and fluid stagnation around the leaflets. This in turn compromises the durability of the BHV and results in an increased probability of thrombi formation or calcification (22, 23). To improve the clinical diagnosis of SVD and enhance the patient's continuum of care, a standardized assessment of BHV hemodynamics has been developed to evaluate the impact of SVD across different BHV designs. Multiple consortiums have established criteria intended to define how changes in hemodynamic function correlate to bioprosthetic valve failure irrespective of the failure mechanism (24).

## 3 The development of a novel tissue processing technique

### 3.1 ADAPT tissue engineering process

**Figure 2:**
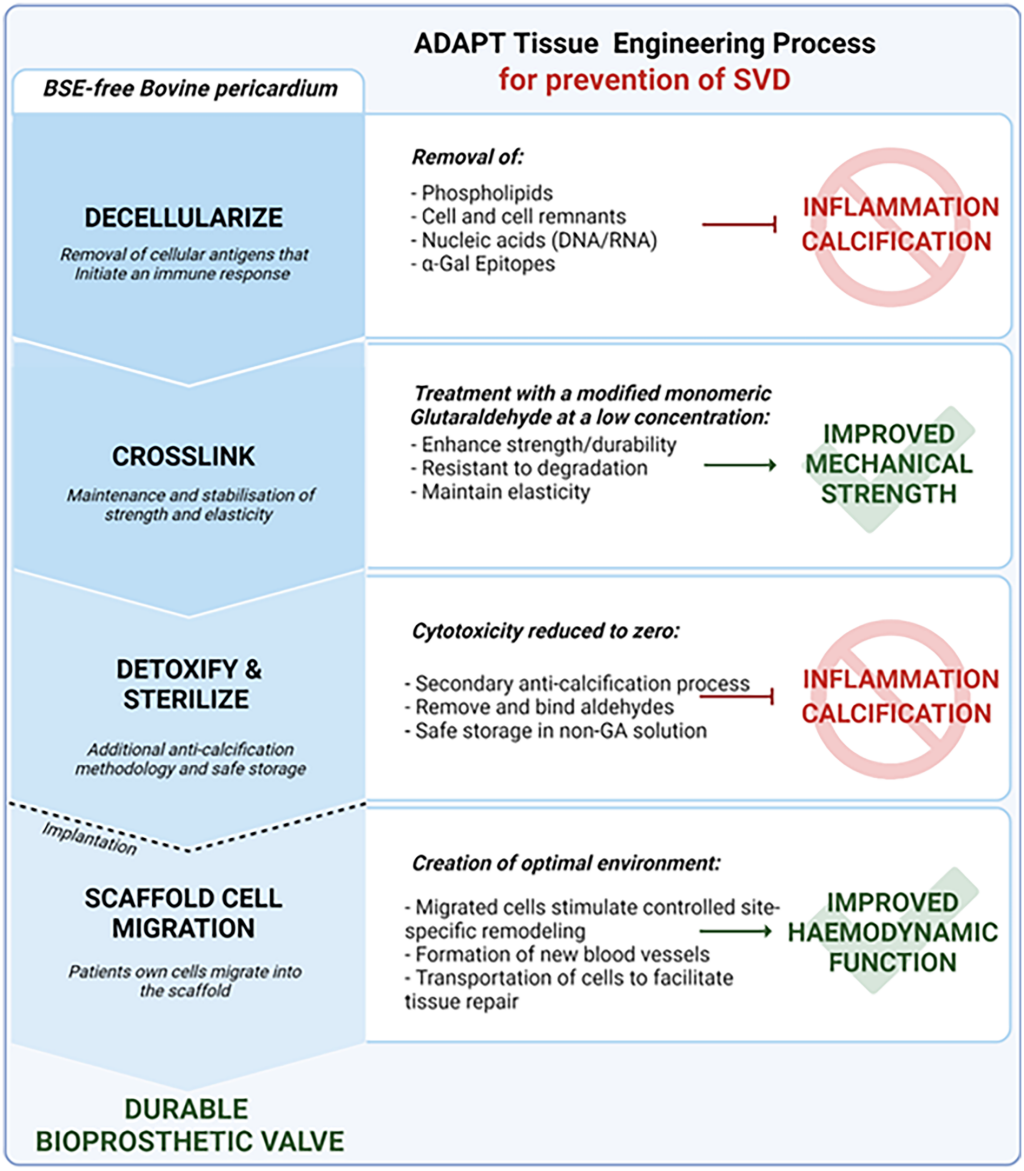
ADAPT™ tissue engineering process. BSE-free bovine pericardium is decellularized to remove all cellular antigens known to initiate inflammation and interrelated calcification mechanisms. The ADAPT™ cross linking treatment with a modified monomeric glutaraldehyde at a low concentration enables maintenance and stabilization of strength and elasticity to improve mechanical resistance. The cytotoxicity is further reduced using detoxification and sterilization processes, as well as a anti-calcification methodology to remove and bind aldehydes and enable safe storage in a non-glutaraldehyde solution. Post-implantation, ADAPT™ tissue provides a scaffold for cell migration to create the optimal environment. Migrated cells can stimulate site-specific remodeling, repair, and enable the formation of new blood vessels to improve hemodynamic function. The ADAPT™ tissue engineering process minimizes risk factors known to cause structural valve deterioration and creates a durable biomaterial optimized for a TAVR application. TAVR, transcatheter aortic valve replacement; SVD, structural valve deterioration.

The ADAPT tissue engineering process (Figure 2) transforms xenograft tissue into durable bioscaffolds that can be used to mimic human tissue for surgical repair in multiple settings. The evolution of the current ADAPT process involved a series of studies that identified and overcame limitations of existing biomaterials and tissue engineering processes resulting in a novel, acellular, biostable and non-calcifying biomaterial.

In 2006, Neethling et al., addressed the limitations of glutaraldehyde-associated calcification by creating a novel anti-calcification tissue engineering processes involving lipid extraction, conformation of collagen structure, and neutralization of residual glutaraldehyde (25). The development of a densely arranged fibrosa with superior tensile and anti-calcification properties reduced the presence of inter-collagen spaces, known to promote the deposition of blood proteins leading to calcification (26). The addition of crosslinking, verified via shrinkage temperature tests and increased resistance to enzymatic degradation (26), was achieved through a modified glutaraldehyde and non-glutaraldehyde sterilization method. This resulted in a significant reduction of calcification compared to conventional glutaraldehyde tissue treatments (9, 27, 28). However, decellularization alone failed to produce a biologically inert matrix (29). In response, a synergized process combining ultra-low glutaraldehyde with the ADAPT anti-calcification process to completely remove all cell remnants was developed and resulted in a significant reduction of residual α-Gal and zero DNA/RNA (30). The advantage of glutaraldehyde-reduction and non-glutaraldehyde storage of bioprosthetic valve tissue was also confirmed in a rabbit model with the Edwards RESILIA™ tissue (31).

While healthy native vasculature tissue and cardiac valves are metabolically active and capable of self-repair, the altered structure in chemically treated biomaterials can prevent the host cell repopulation required to initiate leaflet remodeling, growth, and repair (32). The ADAPT tissue demonstrated successful recellularization by host cells with an absence of a chronic immune response confirmed in a subcutaneous rat model (27) Thus, the attenuation of an immune response, capacity for host cell infiltration with neocapillary formation, and mitigation of calcification suggests that ADAPT processed tissue will perform as well as a clinically implanted biomaterial.

## 4 *In-vitro* and *in-vivo* assessment of ADAPT tissue performance

### 4.1 *In-vitro* testing

The biological properties of ADAPT tissue were compared with several commonly used tissue-engineered bioscaffolds such as bovine pericardial scaffolds, cross-linked with 0.6% glutaraldehyde (XenoLogiX™, PeriGuard®), dye-mediated photo-oxidized (PhotoFix™) and a non-crosslinked porcine scaffold (CorMatrix®) (33). Collectively, ADAPT tissue demonstrated signifficantly higher cross-link stability than non-aldehyde crosslinked competitors, optimised tensile strength without the downfall of stiffness compared to XenoLogiX and PeriGuard and superior biocompatibility with minimal mineralisation potential.

### 4.2 Pre-clinical testing

The replacement of the posterior leaflet of the mitral valve and one of the leaflets the pulmonary valve in growing sheep was used to assess the performance of ADAPT treated tissue *in vivo* (34). Briefly, the mechanical properties of ADAPT tissue were preserved at 7 months post procedure with evidence of a more controlled and gentle healing process than autologous pericardium treated intraoperatively with glutaraldehyde. In addition, the prevention of calcification in this challenging circulatory model suggests that ADAPT treated tissue would be durable and more favorable in a clinical setting. The ADAPT tissue was also extensively tested in a sheep model of complete tri-leaflet aortic valve reconstruction following the Ozaki technique. Aortic valves reconstructed with this pericardial patch demonstrated adequate diastolic function with minimal regurgitation and resistance to calcification. Sustained mechanical integrity of the patch and no calcification were noted, indicating the potential of this material for various valve related pathologies (35).

### 4.3 Clinical use in surgical repairs

The ADAPT-treated BSE-free bovine pericardium has been successfully validated in a Phase II Clinical Trial in pediatric patients with a range of congenital cardiac anomalies. At 6- and 12-months post-implant, no graft related morbidity was reported and echocardiography exhibited intact anatomical and hemodynamically stable repairs without any visible calcification. Subsequent follow up visits at 18–36 months provided no evidence of calcification, infection, thromboembolic events, or graft failure (28). At medium to long term (up to 10 years) follow up, there was no evidence of graft failure, calcification, thromboembolic events, infections, or device-related reinterventions (36). Apart from encouraging results, one of the limitations of this study was the relatively small sample size with only 30 enrolled patients.

Larger scale studies were performed using ADAPT tissue grafts in pediatric patients undergoing surgical repair of congenital heart defects (*n* = 377) in Australia and the United Kingdom (37). In this setting, ADAPT-treated tissue demonstrated excellent durability with no evidence of endocarditis or calcification via echocardiogram or magnetic resonance imaging over a median follow up period of 24 months.

Similarly, in a recent case study of a young adult with symptomatic right heart failure secondary to tricuspid valve regurgitation, ADAPT tissue was reported to have excellent durability and function when used in a complex reconstruction of the anterior tricuspid leaflet (38). Moreover, at the 2 year follow up assessment, the repair remained intact with an improvement in symptoms and complete resolution of hepatic congestion and heart failure.

From a clinical perspective, ADAPT tissue has demonstrated satisfactory durability and elasticity intraoperatively when used a variety of cardiac surgical repairs and reconstructions, providing optimal implantation to patient tissues (39). As expected, no symptoms of pseudoaneurysm, patch thickening or calcification were observed at the patch site at a short term follow up. Additionally, ADAPT treated BSE-free bovine pericardium has been predicted to reduce the incidence of re-operation, increase in quality-adjusted life year after procedure, and reduce costs over a 40-year time horizon relative to xenogeneic and synthetic patches (40). Taken together, these studies and clinical trials confirm that the cellularity, biostability, and non-calcifying properties make the ADAPT tissue process a superior durable biomaterial that can be easily used in surgery (Table 1).

**Table 1:** Incidence of SVD between ADAPT-treated and non-ADAPT treated scaffolds.

Product	SVD rate (%)	Reference
ADAPT-treated		
Xenogeneic scaffold	0.0	Neethling et al. (26–28) Bell et al. (37)
Non-ADAPT treated		
Surgical		
Xenogeneic Scaffolds	18.2	Veličković et al. (40)
Autologous Scaffolds	12.5	Veličković et al. (40)
Synthetic Scaffolds	35.0	Veličković et al. (40)
CoreValve	26.6	Gleason et al. (41)
Mosaic, Epic, Trifecta, Perimount, or Sorin Mitroflow	24	Søndergaard et al. (42)
Transcatheter		
CoreValve, SAPIEN, Portico, or Other	9.1	Blackman et al. (43)
First-generation CoreValve or SAPIEN	14.9	Deutsch et al. (44)
SAPIEN, CoreValve, or Jena	11.2	Durand et al. (45)
CoreValve	4.6	Testa et al. (46)
PVT/Cribier-Edwards, SAPIEN, or SAPIEN XT	3.2	Eltchaninoff et al. (47)
CoreValve	9.5	Gleason et al. (41)
First-generation CoreValve	4.8	Søndergaard et al. (42)

SVD, structural valve deterioration.

## 5 Development of a novel transcatheter heart valve

In addition to using a tissue treatment with maximum protection against extrinsic and intrinsic calcification, optimization of the TAVR leaflet design is imperative to help reduce stress within the BHV leaflet caused during typical valve function. The established TAVR valve design consists of three flexible leaflets attached to a stent via sutures to replciate the semilunar form of the native valve. The attachment of multiple leaflets sewn together can compromise the durability of the TAVR as sutures create a hotspot for increased mechanical tension thereby worsening SVD (20, 22, 32, 48).

Twenty years ago, the first demonstration that 3D leaflet geometry positively influenced leaflet stress distribution and coaptation when compared to a traditional two-dimensional leaflet geometry was published (7). In 2017, building off this experimental finding and inspired by the basic anatomical features and geometry of the native human aortic valve as described by Mercer in 1973 (49), a single piece of ADAPT tissue was molded into a 3D aortic valve and attached securely to a stent with a minimal number of sutures as illustrated previously [Central illustration A (6)]. Within this design, the basic curvature of the leaflet is hemiparaboloid with a parabolic outline in radial cross section. Each leaflet consists of a belly with two enlarged coaptation surfaces (lunulae) to ensure optimal hemodynamics during the cardiac cycle (50). The belly of the cusp is designed to allow for maximum washout of the native sinuses, which lowers the risk associated with thromboemboli formation (51). The coaptation surface areas are significantly increased compared to traditional flat sheet cusp designs, reducing commissural stress and thereby benefitting long-term durability. In addition, the leaftlet design leverages the advantage of the inherent elasticity of ADAPT tissue to withstand the mechanical stresses exerted on the valve throughout the cardiac cycle (9). The thickness and elastic modulus of the leaflet is optimized to avoid leaflet fluttering associated with accelerated fatigue and premature failure of flexible biomaterials (52). Durability is further enhanced through the specific orientation of the pericardium during molding of the valve, which ensures anisotropism to retain the variable elastic modulus of the natural leaflet in the circumferential and radial directions, respectively.

## 6 *In vitro* and *in vivo* assessment of the DurAVR TAVR

Since its inception in 2017, the evolution of the DurAVR TAVR system has progressed rapidly. Leaflet and frame durability has been proven through standardized benchtop methods, such as accelerated wear testing, and testing in chronic ovine models has shown excellent resistance to calcification, fibrosis, and thrombosis. The first human implants were completed in 2022, with encouraging post-implant hemodynamics [Panel B of the Central Illustration in reference (6)] and the presence of near-laminar systolic flow characteristics with 2D cardiac magnetic resonance imaging at 6 months [Panel C of Central Illustration in reference (6)]. Subsequent 1 year follow up data demonstrated a sustained hemodynamic performance indicating valve function was maintained (6). Taken together, this early clinical data suggests that ADAPT tissue combined with the biomimetic 3D single leaflet design is a safe and effective future solution for complete valve replacement, which could position DurAVR as one of the potential preferred choices among the next generation of TAVR valves.

## 7 Discussion

The ADAPT tissue engineering process has greatly improved the biostability and performance of bovine tissue grafts used for surgical repair in the congenital pediatric population. To meet the rising need for a durable TAVR, the ADAPT tissue scaffold has been used to create a biomimetic 3D single piece valve with optimal hemodynamic and durable properties. Early clinical data has shown the restoration of near-laminar flow at 6 months and excellent hemodynamic performance at 1 year. Thus, the combination of the unique design of the DurAVR biomimetic valve with the superior biostability of ADAPT tissue is poised to revolutionize patient management in the treatment of aortic stenosis.
